# Safety Evaluation of Sumizyme PEG: A 90-Day Repeated-Dose Oral Toxicity Study and Comprehensive Genotoxicity Assessment of an Endo-1,3(4)-β-glucanase from *Talaromyces versatilis* PF8

**DOI:** 10.3390/toxics14060458

**Published:** 2026-05-24

**Authors:** Andreas Dietrich, Jürgen Meinl, Lauren Park, Dylan Fronda, Moustafa Kardjadj

**Affiliations:** 1 ERBSLÖH Geisenheim GmbH, Erbslöhstrasse 1, 65366 Geisenheim, Germany; 2 dicentra, Toronto, ON M4W 3E2, Canada

**Keywords:** endo-1,3(4)-β-glucanases, genotoxicity, repeated-dose toxicity, safety assessment

## Abstract

Sumizyme PEG, a glucanase/cellulase enzyme preparation produced by *Talaromyces versatilis* PF8, was investigated to characterize its systemic and genotoxic toxicity profile to support its intended use in food processing applications. A comprehensive toxicological program was conducted in accordance with OECD guidelines, comprising a bacterial reverse mutation (Ames) test, an in vitro chromosomal aberration assay, an in vivo micronucleus test, and a 90-day repeated-dose oral toxicity study in male and female Crl:CD(SD) rats. In the subchronic study, Sumizyme PEG was administered by oral gavage at doses of 107, 1070, and 10,700 U/kg/day. No treatment-related adverse effects were observed across clinical, hematological, biochemical, urinalysis, organ weight, or histopathological endpoints, and the highest dose was identified as the NOAEL. Genotoxic testing showed no consistent mutagenic or clastogenic response across the test battery. A positive in vitro signal was observed in CHL/IU cells; however, this was not reproduced in a human TK6 cell assay or in vivo micronucleus testing, indicating assay-dependent sensitivity within a weight-of-evidence framework. Overall, the integrated dataset does not indicate a consistent treatment-related systemic or genotoxic effect under the conditions of the studies conducted.

## 1. Introduction

Endo-1,3(4)-β-glucanases are cellulolytic enzymes that hydrolyze β-1,3- and β-1,4-glycosidic bonds in glucans, producing soluble oligosaccharides and glucose. Similar to classical cellulose-degrading enzymatic systems such as endo-β-1,4-glucanase (EG), exo-β-1,4-glucanase (CBH), and β-1,4-glucosidase (BG), they act in a coordinated manner to break down polysaccharides. While EG cleaves internal β-1,4 bonds, endo-1,3(4)-β-glucanases preferentially target β-1,3/β-1,4 linkages, generating oligosaccharides for various industrial applications [1,2].

The present study investigates the toxicological profile of Sumizyme PEG, a novel endo-1,3(4)-β-glucanase enzyme preparation derived from *Talaromyces versatilis* PF8. Unlike many industrial enzymes, Sumizyme PEG is derived from a non-genetically modified production organism and is not produced using a recombinant heterologous expression system, resulting in a unique fungal-derived protein matrix. Although various β-glucanase formulations are generally considered safe in food processing, fermentation-derived enzyme concentrates may contain low levels of co-produced proteins, peptides, or other fermentation-related constituents, warranting a detailed toxicological characterization to confirm biological compatibility under the intended conditions of use.

The safety profile was evaluated using an integrated toxicological approach aligned with international OECD test guidelines and principles described by the EFSA and FDA for food enzyme safety evaluation. Specifically, a 90-day repeated-dose oral toxicity study was conducted using male and female Crl:CD(SD) [SPF] rats to determine potential treatment-related systemic effects and establish a no-observed-adverse-effect level (NOAEL) for Sumizyme PEG. In parallel, a battery of in vitro and in vivo genotoxicity assays was conducted to evaluate the potential for gene mutations, structural chromosomal aberrations, and numerical chromosomal alterations.

The objectives of these studies were to comprehensively characterize the systemic and genotoxic toxicity profile of Sumizyme PEG using an integrated toxicological program comprising in vitro and in vivo genotoxicity assays and a 90-day repeated-dose oral toxicity study in rats. These studies were conducted in compliance with OECD guidelines and under conditions relevant to its intended use, with consideration of assay-specific limitations associated with complex enzyme preparations, thereby providing data to support a robust safety evaluation of this enzyme for use in food processing applications.

## 2. Materials and Methods

### 2.1. Test Item and Dose Formulation

The test item, Sumizyme PEG (Lot No. 110607T), is a β-glucanase enzyme preparation derived from *T. versatilis*, provided by Shin Nihon Chemical Co., Ltd. (Anjyo, Aichi, Japan) as a clear, dark brown liquid with a nominal enzyme activity of 1070 U/mL. The substance was stored under frozen conditions (−30 °C to −15 °C). Stability of the test item under the experimental conditions was confirmed through freeze–thaw stability testing cycles prior to study initiation.

Dose formulations were prepared daily by diluting the test item with Water for Injection (Japanese Pharmacopoeia; Otsuka Pharmaceutical Factory, Inc., Tokyo, Japan) to achieve prescribed concentrations. The undiluted test substance (1070 U/mL) was utilized for the high-dose group, while appropriate dilutions were prepared for the lower dose groups. The enzymatic activity of the dose formulations was verified on Days 1, 43, and 83, confirming that concentrations remain within the acceptable range (85.0–115.0% of nominal activity).

### 2.2. Repeated-Dose Toxicity Study (OECD 408)

#### 2.2.1. Study Animals and Husbandry

Male and female Crl:CD(SD) [SPF] rats were sourced from Charles River Laboratories Japan, Inc. (Yokohama, Japan) at 4 weeks of age. Following an 8-day acclimation period, healthy animals were randomized into study groups using a computerized stratification method. At the study initiation of dosing (Day 1), animals were 5 weeks old, with body weights ranging from 154 to 189 g for males and 128 to 151 g for females.

Rats were housed individually in stainless steel wire mesh cages within a barrier facility maintained at 22.8–23.1 °C with a relative humidity of 39.8–64.7%, and a 12 h light/dark cycle. A commercial irradiated diet (CRF-1, Oriental Yeast Co., Ltd., Tokyo, Japan) and tap water were provided ad libitum.

#### 2.2.2. Study Design and Dosing

The study design adhered to OECD Guideline for the Testing of Chemicals 408 (1998). Eighty rats were assigned to four groups (10/sex/group). Based on a previous 2-week dose-finding study in which no toxicity was observed up to 10,700 U/kg, the doses for the 90-day study were set at 107, 1070, and 10,700 U/kg/day. A concurrent control group received the vehicle (Water for Injection) alone.

The test item was administered once daily by oral gavage for 90 consecutive days at a dose volume of 10 mL/kg body weight, adjusted based on the most recent body weight measurement.

#### 2.2.3. Observations and Measurements

**Clinical Signs and Mortality:** Animals were observed for general clinical signs and mortality twice daily (before and after dosing) throughout the administration period.

**Body Weight and Food Consumption:** Individual body weights were recorded on Day 1 and weekly thereafter (Days 8, 15, 22, 29, 36, 43, 50, 57, 64, 71, 78, 85, and 90). Food consumption was quantified over corresponding weekly intervals.

**Ophthalmology:** Ophthalmological examinations were performed on all animals at baseline, and on the control and high-dose (10,700 U/kg) groups on Day 86.

**Urinalysis:** Urinalysis was conducted on all animals on Days 85–86. Fresh urine samples (collected over approx. 17 h) were analyzed for pH, protein, glucose, ketone bodies, bilirubin, occult blood, and urobilinogen. Urine sediment, volume, osmotic pressure, and electrolytes (Na, K, Cl) were also evaluated.

#### 2.2.4. Clinical Pathology

Blood samples were collected from the abdominal aorta under isoflurane anesthesia at scheduled necropsy (Day 91 or 92) following an overnight fasting.

- **Hematology:** Standard parameters including red blood cell count (RBC), hemoglobin (HGB), hematocrit (HCT), mean corpuscular volume (MCV), mean corpuscular hemoglobin (MCH), mean corpuscular hemoglobin concentration (MCHC), reticulocyte ratio, platelet count (PLT), white blood cell count (WBC), and differential leukocyte count (neutrophils, lymphocytes, monocytes, eosinophils, basophils, large unstained cells) were measured.
- **Blood Coagulation:** Prothrombin time (PT), activated partial thromboplastin time (aPTT), and fibrinogen were determined using citrate plasma.
- **Blood Chemistry:** A comprehensive panel including total protein, glucose, triglycerides, total cholesterol, blood urea nitrogen (BUN), creatinine, total bilirubin, aspartate aminotransferase (AST), alanine aminotransferase (ALT), alkaline phosphatase (ALP), gamma-glutamyl transpeptidase (*y*-GTP), calcium, inorganic phosphorus, sodium, potassium, and chloride was analyzed.
- **Serum Protein Electrophoresis:** Albumin ratio and globulin fractions (alpha1, alpha2, beta, gamma) were determined.

#### 2.2.5. Necropsy and Histopathology

All animals underwent a full macroscopic necropsy. Organ weights were recorded for major organs, including the brain, pituitary, thyroids (with parathyroids), thymus, heart, lungs, liver, spleen, kidneys, adrenals, testes, epididymides, prostate, seminal vesicles, ovaries, and uterus.

Histopathological examination was performed on a comprehensive list of tissues from all animals in the control and high-dose (10,700 U/kg) groups. Gross lesions from all groups were also examined. Tissues were fixed in 10% neutral buffered formalin (tests in formalin-acetic acid fixative), embedded in paraffin, sectioned, and stained with hematoxylin and eosin (H&E).

### 2.3. Genotoxicity Studies

#### 2.3.1. Bacterial Reverse-Mutation Assay (Ames Test)

The mutagenic potential of Sumizyme PEG was evaluated per OECD Guideline 471 and GLP standards using *Salmonella typhimurium* strains TA100, TA98, TA1535, and TA1537, and *Escherichia coli* WP2uvrA. Preliminary dose-finding studies demonstrated accelerated growth of the background bacterial law. This observation suggests the presence of nutritive components, such as free amino acids (e.g., histidine), within the enzymatic preparation which may facilitate supplemental cell divisions independent of mutagenic activity [3,4,5]. To isolate the genetic assessment from potential nutritional interference, a modified Induced Mutation Frequency (IMF) method was implemented for strains TA100, TA98, and TA1535. Under this modified protocol, bacteria were washed by centrifugation after a 60 min pre-incubation with the test substance to remove the exogenous matrix. This step ensured that the colony counts reflected true reversion events rather than nutrient-stimulated growth.

Doses for the main study included levels up to 107.00 U/plate (equivalent to 100 µL of stock solution). Assays were conducted with or without metabolic activation (+S9/−S9). Triplicate plates were used for dose-finding and duplicate plates for the main study.

#### 2.3.2. In Vitro Mammalian Chromosomal Aberration Test

An in vitro chromosomal aberration assay was conducted using the Chinese hamster lung fibroblast cell line (CHL/IU) according to OECD Guideline 473. Cells were exposed to Sumizyme PEG in three treatment series: short-term (6 h) with and without metabolic activation (±S9), and continuous (24 h) without S9.

Based on preliminary cell growth inhibition tests, dose levels were set at 13.4, 26.8, 53.5, and 107 U/mL. Structural aberrations were scored for 200 metaphases per concentration. A confirmatory study was subsequently performed for the −S9 short-term and 24 h continuous treatments using doses of 36.7, 52.4, 74.9, and 107 U/mL to verify reproducibility and dose-dependency.

#### 2.3.3. In Vitro Mammalian Cell Micronucleus Test

The micronucleus-forming potential was assessed in TK6 human lymphoblastoid cells following OECD Guideline 487. Cytotoxicity was measured by relative population doubling (RPD). Micronuclei (MN) were scored in a minimum of 2000 mononuclear cells per concentration.

#### 2.3.4. In Vivo Mammalian Erythrocyte Micronucleus Test

An in vivo micronucleus assay was conducted in male Crl:CD(SD) rats in accordance with OECD Guideline 474. The maximum dose was set at 10,700 U/kg, administered via oral gavage once daily for two consecutive days. Bone marrow samples were collected from the femur 24 h after the final dose (*n* = 5 animals per group). The frequency of micronucleated immature erythrocytes (MNIEs) was determined by scoring 2000 immature erythrocytes (IE) per animal. Bone marrow cytotoxicity was assessed by the IEs to total erythrocytes ratio.

### 2.4. Statistical Analysis

Data were assessed for homogeneity of variance using Bartlett’s test. Homogeneous data were analyzed using Dunnett’s multiple comparison test, while heterogeneous data were analyzed using Steel’s test to compare treatment groups against the control. Fisher’s exact test was used for survival rates, and Wilcoxon’s test was used for histopathological findings. The probability value of *p* < 0.05 was considered statistically significant.

For the bacterial reverse-mutation assay, a result was considered positive if the number of revertant colonies increased to at least twice that of the negative control in a dose-dependent or reproducible manner; no formal statistical test was applied as per standard practice for this assay.

For the in vitro chromosome aberration test and the in vitro micronucleus assay, the incidence of cells with aberrations or micronuclei was compared between the treated and negative control groups using Fisher’s exact test (one-sided, *p* < 0.025). Additionally, the Cochran–Armitage trend test was utilized to evaluate dose-dependency (*p* < 0.05).

For the in vivo micronucleus assay, the frequency of micronucleated immature erythrocytes (MNIEs) was analyzed using the Kastenbaum and Bowman conditional binomial test (*p* < 0.025). The ratio of immature erythrocytes to total erythrocytes (IE/TE), used as an index of bone marrow toxicity, was analyzed for homogeneity of variance using Bartlett’s test, followed by Dunnett’s test or the Steel’s test for group comparisons (*p* < 0.05).

## 3. Results

### 3.1. General Observations, Body Weight, and Food Consumption

No mortality occurred during the study period. Daily clinical observations revealed no treatment-related abnormalities in any dose group. Mean body weights and body weight gains in both sexes were comparable to controls throughout the 90-day administration period. No statistically significant differences were noted between the Sumizyme PEG-treated groups and the control group at any time point (Table 1). Food consumption remained unaffected by treatment, with no statistically significant differences noted in males. A transient increase in food consumption was noted in low-dose females (107 U/kg) during the final week; however this was considered biologically inconsequential due to the lack of dose-dependency (Table 2).

### 3.2. Clinical Pathology

#### 3.2.1. Hematology and Coagulation

Hematological assessment revealed no adverse effects of the treatment. A statistically significant increase in the neutrophil ratio was observed in males in the 107 U/kg group; however, this finding was interpreted as incidental, as it lacked dose-dependency. All other parameters were comparable to controls (Table 3 and Table 4).

#### 3.2.2. Blood Chemistry

Serum biochemical analysis indicated mild variations in electrolytes and proteins in the high-dose group (Table 5). Males in the 10,700 U/kg group exhibited statistically significant decreases in sodium (143.7 vs. 144.8 mmol/L) and chloride (106.4 vs. 108.6 mmol/L) levels compared to controls. Females in the 10,700 U/kg group showed significantly lower total protein (6.36 vs. 6.70 g/dL) and albumin (3.40 vs. 3.77 g/dL). These changes were considered toxicologically non-significant as the magnitudes were marginal and values remained within histological backgrounds ranges.

Although females in the high-dose group showed numerically higher mean AST and ALT values, these were driven by a single outlier (Animal No. 2307) and did not reach statistical significance. The absence of associated adverse histopathological findings suggests these biochemical variations lack clinical relevance.

#### 3.2.3. Urinalysis

No treatment-related changes were observed in urinalysis parameters (Table 6). A statistically significant increase in osmotic pressure and sodium excretion was noted in low-dose females (107 U/kg), which was attributed to individual biological variation given the absence of a dose-dependent response.

### 3.3. Ophthalmology and Organ Weights

Ophthalmological examinations conducted on Day 86 revealed no abnormalities attributable to Sumizyme PEG. Findings such as corneal opacity and particulate lens opacity were noted in both control and high-dose groups, consistent with spontaneous age-related changes.

Regarding organ weights, statistically significant increases in absolute brain weight (2.31 ± 0.09 g vs. 2.22 ± 0.08 g) and absolute prostate weight (1711 ± 113 mg vs. 1418 ± 227 mg) were observed in mid-dose males (1070 U/kg) (Table 7). Additionally, a statistically significant decrease in relative liver weight (2.23 ± 0.17 g/100 g vs. 2.36 ± 0.14 g/100 g) was noted in mid-dose females (Table 8). These findings were deemed to lack toxicologically importance as they were not observed in the high-dose (10,700 U/kg) group and lack corresponding histopathological or clinical chemistry alterations.

### 3.4. Macroscopic and Histopathological Evaluation

Macroscopic examination revealed no treatment-related changes in any group. Histopathological evaluation of the control and high-dose (10,700 U/kg) groups showed no lesions associated with Sumizyme PEG administration. Observed findings, such as focal microgranuloma in the liver or hyaline droplets in the kidneys, were characterized as spontaneous background lesions. One female in the high-dose group showed isolated elevations in AST and ALT (Table 5), but the absence of corresponding histological hepatocellular changes indicates a lack of organ toxicity.

### 3.5. Genotoxicity Evaluation

#### 3.5.1. Ames Bacterial Reverse-Mutation Assay

In the preliminary and initial standard assays, Sumizyme PEG was associated with a growth-promoting effect on the background bacterial lawn, accompanied by increases in revertant colonies in strains TA100, TA98, and TA1535. This phenomenon is consistent with established literature regarding enzymatic preparations containing free amino acids, which can facilitate supplemental bacterial growth and colony formation independent of mutagenic activity [3,4,5]. To isolate the mutagenic assessment from this nutritional matrix interference, a modified IMF method incorporating centrifugal washing steps was implemented. By removing exogenous low-molecular weight components prior to plating, the assay targeted the intrinsic genomic stability of the test item. Following this protocol, no biologically or statistically significant increases in revertant colonies were observed in any tester strain (TA100, TA98, TA1535, TA1537, or *E. coli* WP2uvrA) at doses up to 107 U/plate, either with or without S9 metabolic activation (Table 9). The positive controls induced marked increases in revertant colonies, confirming the sensitivity and validity of the system.

#### 3.5.2. In Vitro Chromosomal Aberration Assay

The clastogenic potential of Sumizyme PEG was evaluated in Chinese hamster lung (CHL/IU) cells (Exp. No. E377), with findings summarized in Table 10. In the short-term treatment (6 h) without metabolic activation (−S9), a statistically significant increase in the frequency of cells with structural chromosomal aberrations was observed at 107 U/mL. This effect was reproducible in the confirmative study, where the aberration rate reached 9.5% (*p* < 0.025) compared to 1.0% in the concurrent solvent control. Under continuous exposure conditions (24 h) without S9, a dose-dependent increase in structural aberrations was observed. The frequency of aberrant cells increased significantly starting at 53.5 U/mL (11.0%) and reached 25.5% at the 107 U/mL in the confirmative phase (*p* < 0.025). In the presence of metabolic activation (+S9), no statistically significant increases in structural chromosomal aberrations were observed at any concentration tested; the aberration frequency at 107 U/mL (1.0%) was comparable to that of the negative control. Throughout all treatment conditions, the incidence of polyploid cells remained within normal historical limits (≤0.5%), indicating no increase in numerical chromosomal aberrations.

#### 3.5.3. In Vitro Mammalian Cell Micronucleus Test

The in vitro micronucleus test in TK6 cells demonstrated no evidence of genotoxicity. At concentration up to 23,900 µg/mL, cell viability (RPD) remained above 80% for the 3 h treatments and dropped to 41.4% for the 24 h treatment, indicating appropriate cytotoxicity for evaluation. The frequency of micronuclei in treated cultures was comparable to the vehicle controls and fell within historical control ranges across all treatment schedules (Table 11).

#### 3.5.4. In Vivo Mammalian Erythrocyte Micronucleus Test (Analytics Summary)

The in vivo assessment (Exp. No. E693) confirmed the lack of systemic genotoxicity. All dose groups showed bone marrow proliferation ratios (IE/TE) and MNIE frequencies that were statistically indistinguishable from the vehicle control (Table 12).

## 4. Discussion

### 4.1. Repeated-Dose Toxicity Evaluation

The 90-day repeated-dose oral toxicity study in Crl:CD(SD) [SPF] rats demonstrates that Sumizyme PEG is well tolerated at all tested doses of 107, 1070, and 10,700 U/kg/day, with no mortality or clinical signs indicative of systemic toxicity in either sex. Body weight trajectories, weight gain, and food consumption remained comparable to controls, and transient increases in food intake observed in low-dose females during the final week were non-dose dependent and consistent with normal biological variability. These findings indicate that Sumizyme PEG does not produce overt systemic toxicity under the conditions of this study.

Hematological and coagulation parameters were not adversely affected. A statistically significant increase in neutrophil ratio in mid-dose males was within historical control ranges and lacked a dose–response relationship, suggesting limited toxicological relevance under the conditions of this study. Red blood cell indices, leukocyte profiles, and coagulation markers were comparable to controls, indicating that repeated oral exposure to Sumizyme PEG did not produce hematologic or immune changes under the study conditions. Serum biochemistry revealed minor variations in electrolytes and protein levels at the highest dose. Mild reductions (*p* < 0.05) in sodium in males and total protein in females, as well as modest decreases (*p* < 0.01) in chloride in males and albumin in females, were non-dose dependent, remained within historical control ranges, and were not associated with histopathological alterations, supporting their interpretation as biological variability rather than treatment-related effects. Occasional elevations in AST and ALT in individual animals were not statistically significant and were uncorrelated with liver morphology, supporting the absence of hepatotoxicity. Urinalysis parameters, including volume, pH, protein, glucose, and sediment, remained comparable to controls. Isolated increases in osmotic pressure, sodium, and sodium excretion in low-dose females were considered of limited toxicological relevance due to the absence of a dose–response relationship, lack of histopathological correlation, and consistency with normal biological variation. Ophthalmologic examinations did not indicate treatment-related abnormalities, and corneal and lens changes observed in both control and high-dose groups were consistent with age-related spontaneous findings in rats. Significant changes in organ weights included increased absolute brain (*p* < 0.05) and decreased absolute prostate (*p* < 0.05) in mid-dose males and reduced relative liver in mid-dose females. These findings lacked a dose-dependency, were absent at the high dose, and were not accompanied by histopathological or functional alterations, suggesting no biologically meaningful relationship to Sumizyme PEG administration. Macroscopic and microscopic evaluations revealed no treatment-related lesions. Background findings, such as hepatic microgranulomas and renal hyaline droplets, occurred at similar incidences in controls and treated animals and are consistent with spontaneous pathology in this strain of rats. Taken together, the absence of consistent dose-dependent changes across clinical pathology, organ weight, and histopathological endpoints indicates no evidence of biologically meaningful systemic toxicity under the conditions of this study.

Overall, repeated oral administration of Sumizyme PEG did not induce systemic, hematologic, metabolic, renal, hepatic, ocular, or morphological toxicity. Minor variations observed in select parameters were non-dose dependent and within the range of normal biological variability. The highest tested dose of 10,700 U/kg/day was identified as the NOAEL. These findings, considered within the limitations of a 90-day rodent study and under conditions of intended oral exposure, support a substantial margin of safety for Sumizyme PEG.

### 4.2. Genotoxicity Evaluation

Sumizyme PEG was evaluated for genotoxic potential using a battery of in vitro and in vivo assays. In bacterial reverse-mutation assays, increases in revertant colonies were observed in strain TA1535 in the absence of metabolic activation. This response may indicate base-pair substitution activity under these specific conditions. One possible explanation is that free amino acids present in the enzyme preparation contributed to apparent growth stimulation, an effect reported for nutrient-rich substances [3,4,5]; however, this mechanism was not directly investigated in the present study. The lack of a corresponding response in the presence of S9 and the absence of increases following application of the Induced Mutation Frequency (IMF) protocol designed to reduce potential interference suggest that the observed effect may be influenced by assay-related factors. However, in the absence of targeted mechanistic controls, a definitive attribution cannot be made.

Sumizyme PEG induced structural, but not numerical, chromosome aberrations in CHL/IU cells under the conditions of this study, indicating a positive response in this in vitro system. However, in vitro chromosome aberration assays in CHL/IU cells have been reported to yield positive results that may be influenced by cell-line-specific characteristics, including differences in DNA damage response and repair capacity [6,7]. Accordingly, these results should be interpreted as a positive in vitro signal within the context of assay limitations, rather than definitive evidence of in vivo genotoxicity.

To further address the biological relevance of these findings, Sumizyme PEG was assessed in human TK6 cells and in an in vivo micronucleus assay, which provided complementary endpoints for detecting both structural and numerical chromosomal aberrations under more physiologically relevant conditions [8,9]. In a follow-up in vitro micronucleus assay using human-derived p53-competent TK6 cells, no significant increases in micronuclei were observed across any treatment conditions. This suggests that the positive response observed in CHL/IU cells may be dependent on cell-line-specific sensitivity rather than a general genotoxic effect; however, differences in assay design and endpoint sensitivity should also be considered.

In vivo testing confirmed that Sumizyme PEG does not induce chromosomal damage under the conditions of the assay. In the SD rat bone marrow micronucleus assay, no increases in micronucleated erythrocytes or changes in the immature-to-total erythrocyte ratio were observed at any dose, indicating no evidence of chromosomal damage in vivo under the study conditions. Furthermore, a lack of in vivo chromosomal damage has been reported for other endo-1,3(4)-β-glucanase enzymes from fungal sources [10,11]. Taken together, the dataset shows a positive response in one in vitro assay (CHL/IU chromosomal aberration test), which was not reproduced in human cell-based or in vivo system. Within a weight-of-evidence framework, these findings do not indicate a consistent genotoxic effect under the conditions tested. However, the in vitro signals observed in bacterial and CHL/IU systems should be considered as part of the overall uncertainty and may warrant further mechanistic investigation.

### 4.3. Perspectives and Limitations

The highest tested dose of Sumizyme PEG (10,700 U/kg/day) produced no treatment-related adverse effects and was therefore identified as the NOAEL. However, this 90-day subchronic study was conducted in a single rodent species following oral administration and does not address potential effects associated with long-term (chronic), reproductive, or developmental exposure. In addition, the evaluation was limited to the specific dose range tested and did not include mechanistic investigations of observed in vitro findings. Importantly, a conservative dietary exposure assessment was performed based on intended use levels of the enzyme in food processing, using a Total Theoretical Maximum Daily Intake (TMDI) approach. This model-based estimate reflects a worst-case exposure scenario rather than measured dietary intake and was used to derive a quantitative margin of safety in relation to the NOAEL. While these findings support interpretation within a safety assessment framework, the exposure estimate remains model-based and may not fully reflect a real-world dietary exposure scenario.

Furthermore, positive findings in CHL/IU cells highlight the presence of a reproducible in vitro signal in one assay system and underscore the importance of interpreting genotoxicity results across multiple test models, including in vivo assays. The divergence between in vitro assay outcomes suggests assay-dependent sensitivity that warrants consideration within a weight-of-evidence framework rather than isolated endpoint interpretation. Taken together, the overall dataset does not indicate a consistent systemic or genotoxic hazard under the conditions of the studies conducted.

## 5. Conclusions

This study evaluated the safety profile of Sumizyme PEG through a 90-day repeated-dose oral toxicity study in rats and a comprehensive battery of in vitro and in vivo genotoxicity assays. Sumizyme PEG was well tolerated in rats at all tested doses, including up to 10,700 U/kg/day, with no treatment-related adverse effects observed across systemic toxicological endpoints, and the NOAEL was identified as the highest dose tested. Genotoxicity testing, including Ames, in vitro chromosomal aberration, micronucleus assays, and the in vivo rat bone marrow micronucleus assays, did not demonstrate a consistent mutagenic or clastogenic response across the test battery. A positive in vitro response in CHL/IU cells was observed but was not reproduced in other tested systems, supporting interpretation within a weight-of-evidence framework and suggesting assay-dependent sensitivity. Taken together, the results across complementary in vitro and in vivo systems do not indicate a consistent treatment-related systemic or genotoxic effect under the conditions of the studies conducted. Study limitations include the 90-day duration, the absence of long-term or reproductive toxicity evaluation, and limited mechanistic investigation of the observed in vitro responses.

## Figures and Tables

**Table 1:** Body weight and body weight gain of rats treated with Sumizyme PEG (Day 90).

Group (U/kg)	Sex	Initial Body Weight (g)	Terminal Body Weight (g)	Body Weight Gain (g)
**Control (0)**	M	140 ± 7	542 ± 29	350 ± 28
	F	140 ± 8	302 ± 31	162 ± 28
**107**	M	140 ± 7	548 ± 55	375 ± 52
	F	140 ± 7	313 ± 16	173 ± 17
**1070**	M	140 ± 7	581 ± 66	409 ± 64
	F	140 ± 11	310 ± 38	170 ± 34
**10,700**	M	140 ± 7	570 ± 64	398 ± 62
	F	140 ± 12	309 ± 23	169 ± 21

Values represent Mean ± S.D. (*n* = 10). No significant differences vs. control.

**Table 2:** Mean daily food consumption (g/day) at selected intervals.

Period (Days)	Sex	Control (0 U/kg)	107 U/kg	1070 U/kg	10,700 U/kg
**Days 1–8**	M	20.3 ± 1.5	20.7 ± 1.4	20.9 ± 1.5	20.5 ± 1.2
	F	14.5 ± 1.3	14.9 ± 1.0	15.0 ± 1.2	14.7 ± 1.4
**Days 43–50**	M	26.5 ± 1.6	26.3 ± 2.6	27.6 ± 2.4	26.9 ± 1.9
	F	17.5 ± 2.0	18.2 ± 0.9	17.7 ± 1.6	17.4 ± 1.6
**Days 85–90**	M	25.4 ± 2.4	25.1 ± 3.1	26.5 ± 2.4	25.8 ± 1.9
	F	16.1 ± 1.6	17.8 ± 1.0 *	17.1 ± 1.9	16.3 ± 1.5

Values represent Mean ± S.D. (*n* = 10). * Significantly different from control (*p* < 0.05). Increased consumption in low-dose females was transient and not dose-dependent.

**Table 3:** Complete hematology profile in male and female rats (Day 91/92).

Parameter (Unit)	Sex	Control	107 U/kg	1070 U/kg	10,700 U/kg
**RBC** (10^6^/uL)	M	8.54 ± 0.48	8.47 ± 0.38	8.37 ± 0.49	8.45 ± 0.45
	F	8.01 ± 0.35	8.24 ± 0.44	8.05 ± 0.20	7.95 ± 0.37
**HGB** (g/dL)	M	15.7 ± 0.6	15.5 ± 0.6	15.2 ± 0.5	15.3 ± 0.6
	F	15.2 ± 0.4	15.1 ± 0.5	15.4 ± 0.2	14.9 ± 0.4
**HCT** (%)	M	44.7 ± 1.7	44.0 ± 1.6	43.7 ± 1.5	43.8 ± 1.6
	F	42.6 ± 1.1	43.3 ± 1.6	43.7 ± 0.8	42.0 ± 1.3
**MCV** (um^3^)	M	52.4 ± 1.9	52.1 ± 2.1	52.3 ± 1.8	51.9 ± 1.7
	F	53.2 ± 1.8	52.6 ± 2.7	54.4 ± 1.6	52.9 ± 2.0
**MCH** (pg)	M	18.4 ± 0.7	18.3 ± 0.6	18.3 ± 0.8	18.1 ± 0.6
	F	19.0 ± 0.4	18.6 ± 0.9	19.2 ± 0.6	18.8 ± 0.7
**MCHC** (%)	M	35.2 ± 0.5	35.2 ± 0.7	34.9 ± 0.3	34.9 ± 0.3
	F	35.7 ± 0.6	35.5 ± 0.4	35.3 ± 0.4	35.5 ± 0.3
**Reticulocyte** (%)	M	2.2 ± 0.5	2.3 ± 0.5	2.3 ± 0.5	2.0 ± 0.3
	F	2.2 ± 0.4	2.3 ± 0.5	2.2 ± 0.6	2.0 ± 0.4
**PLT** (10^3^/uL)	M	1004 ± 132	977 ± 92	991 ± 130	1006 ± 77
	F	1063 ± 147	1029 ± 89	980 ± 57	988 ± 127
**WBC** (10^3^/uL)	M	9.30 ± 2.25	9.08 ± 1.83	8.80 ± 2.55	8.72 ± 1.52
	F	5.29 ± 1.55	4.15 ± 1.27	4.93 ± 1.12	5.75 ± 1.53
**Neutrophil** (%)	M	13.3 ± 2.6	17.6 ± 3.3 *	13.8 ± 4.6	16.4 ± 6.4
	F	16.2 ± 4.8	18.0 ± 4.9	17.7 ± 5.8	16.7 ± 7.1
**Lymphocyte** (%)	M	81.9 ± 3.1	77.4 ± 3.5	81.1 ± 4.7	78.3 ± 6.3
	F	78.3 ± 5.4	76.3 ± 5.5	76.8 ± 6.1	78.1 ± 7.9
**Monocyte** (%)	M	1.9 ± 0.8	2.0 ± 0.5	2.2 ± 0.6	2.5 ± 0.9
	F	2.2 ± 0.8	2.3 ± 0.4	2.4 ± 0.6	2.1 ± 0.9
**Eosinophil** (%)	M	1.4 ± 0.4	1.6 ± 0.5	1.6 ± 0.8	1.5 ± 0.7
	F	1.9 ± 0.5	2.3 ± 1.0	1.9 ± 0.6	1.9 ± 0.5
**Basophil** (%)	M	0.1 ± 0.1	0.1 ± 0.0	0.2 ± 0.1	0.1 ± 0.1
	F	0.1 ± 0.1	0.1 ± 0.1	0.1 ± 0.0	0.1 ± 0.1
**LUC** (%)	M	1.4 ± 0.5	1.3 ± 0.5	1.1 ± 0.4	1.2 ± 0.4
	F	1.3 ± 0.5	1.1 ± 0.3	1.1 ± 0.3	1.2 ± 0.6

Values represent Mean ± S.D. (*n* = 10). * Significantly different from control (*p* < 0.05). RBC: red blood cell; HGB: hemoglobin; HCT: hematocrit; MCV: mean corpuscular volume; MCH: mean corpuscular hemoglobin; MCHC: mean corpuscular hemoglobin concentration; PLT: platelet; WBC: white blood cell; LUC: large unstained cell.

**Table 4:** Blood coagulation parameters in male and female rats (Day 91/92).

Parameter (Unit)	Sex	Control	107 U/kg	1070 U/kg	10,700 U/kg
**PT** (s)	M	12.9 ± 2.5	13.1 ± 1.9	13.2 ± 3.2	12.6 ± 2.0
	F	8.4 ± 0.2	8.6 ± 0.2	8.5 ± 0.3	8.5 ± 0.2
**APTT** (s)	M	27.2 ± 3.5	26.1 ± 2.9	25.8 ± 2.6	26.7 ± 2.8
	F	18.4 ± 1.1	18.5 ± 1.1	18.9 ± 1.7	18.3 ± 1.6
**Fibrinogen** (mg/dL)	M	291 ± 27	285 ± 24	313 ± 42	299 ± 48
	F	221 ± 12	222 ± 23	221 ± 16	244 ± 61

Values represent Mean ± S.D. (*n* = 10). No significant differences vs. control.

**Table 5:** Complete blood chemistry profile in male and female rats (Day 91/92).

Parameter (Unit)	Sex	Control	107 U/kg	1070 U/kg	10,700 U/kg
**Total Protein** (g/dL)	M	6.12 ± 0.22	5.93 ± 0.28	5.97 ± 0.28	5.92 ± 0.28
	F	6.70 ± 0.28	6.42 ± 0.33	6.49 ± 0.24	6.36 ± 0.29 *
**Albumin** (g/dL)	M	2.99 ± 0.15	2.89 ± 0.20	2.97 ± 0.12	2.87 ± 0.09
	F	3.77 ± 0.28	3.51 ± 0.26	3.69 ± 0.15	3.40 ± 0.33 **
**Glucose** (mg/dL)	M	164 ± 16	163 ± 18	165 ± 14	180 ± 15
	F	155 ± 18	157 ± 9	157 ± 19	161 ± 17
**Triglycerides** (mg/dL)	M	60.2 ± 18.4	54.5 ± 25.8	79.0 ± 28.8	61.7 ± 28.7
	F	41.4 ± 22.9	25.8 ± 12.5	35.5 ± 11.9	31.8 ± 15.9
**Total Cholesterol** (mg/dL)	M	71 ± 21	66 ± 9	73 ± 21	70 ± 12
	F	77 ± 11	67 ± 11	69 ± 11	65 ± 16
**BUN** (mg/dL)	M	13.2 ± 2.5	12.5 ± 1.4	13.9 ± 1.6	13.7 ± 1.2
	F	14.6 ± 2.0	15.1 ± 2.1	16.1 ± 3.2	15.6 ± 2.2
**Creatinine** (mg/dL)	M	0.30 ± 0.05	0.27 ± 0.04	0.30 ± 0.02	0.28 ± 0.03
	F	0.30 ± 0.04	0.33 ± 0.06	0.32 ± 0.04	0.32 ± 0.07
**Total Bilirubin** (mg/dL)	M	0.05 ± 0.01	0.04 ± 0.01	0.05 ± 0.02	0.04 ± 0.01
	F	0.06 ± 0.03	0.06 ± 0.02	0.07 ± 0.02	0.06 ± 0.01
**AST** (U/L)	M	84 ± 23	77 ± 17	73 ± 11	84 ± 26
	F	90 ± 20	77 ± 15	85 ± 24	124 ± 150
**ALT** (U/L)	M	28 ± 8	26 ± 3	29 ± 7	29 ± 8
	F	26 ± 7	21 ± 3	24 ± 8	46 ± 70
**ALP** (U/L)	M	283 ± 70	305 ± 42	312 ± 48	315 ± 99
	F	180 ± 50	181 ± 70	179 ± 63	159 ± 52
**Gamma-GTP** (U/L)	M	0.5 ± 0.1	0.6 ± 0.2	0.5 ± 0.2	0.5 ± 0.2
	F	0.9 ± 0.3	0.7 ± 0.2	0.6 ± 0.2	0.8 ± 0.2
**Calcium** (mg/dL)	M	9.43 ± 0.29	9.51 ± 0.32	9.58 ± 0.31	9.45 ± 0.30
	F	9.80 ± 0.34	9.64 ± 0.44	9.80 ± 0.17	9.85 ± 0.34
**Inorganic Phosphorus** (mg/dL)	M	5.69 ± 0.62	5.61 ± 0.38	5.63 ± 0.56	5.92 ± 0.36
	F	4.92 ± 0.79	4.84 ± 1.05	4.74 ± 0.69	5.03 ± 0.78
**Sodium** (mmol/L)	M	144.8 ± 1.0	144.2 ± 1.2	144.1 ± 0.5	143.7 ± 0.8 *
	F	143.7 ± 1.1	144.0 ± 0.5	144.2 ± 0.7	143.8 ± 1.2
**Potassium** (mmol/L)	M	4.66 ± 0.24	4.68 ± 0.21	4.59 ± 0.28	4.78 ± 0.21
	F	4.28 ± 0.26	4.19 ± 0.23	4.32 ± 0.23	4.31 ± 0.24
**Chloride** (mmol/L)	M	108.6 ± 1.2	108.7 ± 1.3	108.2 ± 1.4	106.4 ± 0.8 **
	F	109.3 ± 1.2	109.3 ± 1.2	110.0 ± 1.1	109.1 ± 1.1

Values represent Mean ± S.D. (*n* = 10). * Significantly different from control (*p* < 0.05), ** (*p* < 0.01).

**Table 6:** Quantitative urinalysis parameters in male and female rats (Day 86).

Parameter (Unit)	Sex	Control	107 U/kg	1070 U/kg	10,700 U/kg
**Volume** (mL/17 h)	M	15.8 ± 2.0	16.0 ± 2.5	16.8 ± 3.9	16.0 ± 2.5
	F	8.8 ± 2.0	9.1 ± 1.9	7.6 ± 2.0	8.7 ± 1.6
**Osmotic Pressure** (mOsm/kg)	M	633 ± 96	644 ± 158	609 ± 108	706 ± 140
	F	673 ± 81	818 ± 177 *	771 ± 107	701 ± 110
**Sodium** (mmol/L)	M	22.9 ± 6.5	27.2 ± 8.5	20.1 ± 6.5	23.0 ± 9.7
	F	29.6 ± 6.5	38.9 ± 7.4 *	26.5 ± 9.3	32.5 ± 10.1
**Potassium** (mmol/L)	M	85.2 ± 15.1	83.6 ± 26.2	82.5 ± 16.0	85.6 ± 20.2
	F	75.1 ± 13.7	92.4 ± 27.2	80.6 ± 9.3	72.7 ± 12.8
**Chloride** (mmol/L)	M	27.9 ± 7.5	26.5 ± 6.5	23.1 ± 6.8	27.6 ± 11.2
	F	28.8 ± 10.7	37.0 ± 11.7	25.5 ± 8.6	20.6 ± 3.8
**Sodium Excretion** (mmol/17 h)	M	0.36 ± 0.09	0.44 ± 0.16	0.34 ± 0.11	0.37 ± 0.17
	F	0.26 ± 0.06	0.35 ± 0.10 *	0.20 ± 0.09	0.29 ± 0.14
**Potassium Excretion** (mmol/17 h)	M	1.33 ± 0.16	1.30 ± 0.32	1.35 ± 0.20	1.37 ± 0.40
	F	0.65 ± 0.13	0.81 ± 0.13	0.61 ± 0.16	0.64 ± 0.19
**Chloride Excretion** (mmol/17 h)	M	0.43 ± 0.10	0.42 ± 0.09	0.38 ± 0.07	0.43 ± 0.10
	F	0.25 ± 0.08	0.33 ± 0.10	0.19 ± 0.11	0.18 ± 0.08

Values represent Mean ± S.D. (*n* = 10). * Significantly different from control (*p* < 0.05).

**Table 7:** Absolute organ weights of male and female rats (Day 91/92).

Organ (g)	Sex	Control	107 U/kg	1070 U/kg	10,700 U/kg
**Brain** (g)	M	2.22 ± 0.08	2.28 ± 0.04	2.31 ± 0.09 *	2.27 ± 0.04
	F	2.10 ± 0.08	2.14 ± 0.07	2.09 ± 0.09	2.09 ± 0.06
**Heart** (g)	M	1.52 ± 0.16	1.56 ± 0.18	1.54 ± 0.11	1.54 ± 0.21
	F	0.95 ± 0.09	0.97 ± 0.06	0.94 ± 0.11	0.94 ± 0.09
**Liver** (g)	M	12.40 ± 1.35	13.09 ± 2.49	14.18 ± 2.55	14.56 ± 2.43
	F	7.05 ± 0.79	7.02 ± 0.40	6.84 ± 0.93	7.28 ± 0.57
**Kidneys** (g)	M	3.06 ± 0.22	3.25 ± 0.43	3.31 ± 0.35	3.35 ± 0.39
	F	1.87 ± 0.16	1.92 ± 0.15	1.88 ± 0.24	1.89 ± 0.16
**Spleen** (g)	M	0.79 ± 0.09	0.83 ± 0.10	0.82 ± 0.12	0.86 ± 0.11
	F	0.50 ± 0.07	0.51 ± 0.10	0.50 ± 0.07	0.54 ± 0.09
**Adrenals** (mg)	M	58 ± 10	55 ± 12	59 ± 10	63 ± 11
	F	65 ± 7	73 ± 10	61 ± 10	69 ± 15
**Testes**	M	3.36 ± 0.24	3.41 ± 0.21	3.40 ± 0.13	3.52 ± 0.30
**Ovaries** (mg)	F	86 ± 15	94 ± 18	82 ± 8	97 ± 19
**Thyroid** (mg)	M	25 ± 4	29 ± 6	28 ± 5	29 ± 6
	F	24 ± 6	22 ± 5	23 ± 5	22 ± 5
**Pituitary** (mg)	M	14 ± 2	15 ± 3	15 ± 2	13 ± 2
	F	17 ± 4	16 ± 2	17 ± 4	16 ± 3
**Thymus** (mg)	M	361 ± 89	362 ± 115	349 ± 65	351 ± 120
	F	253 ± 72	268 ± 77	283 ± 59	288 ± 53
**Prostate** (mg)	M	1418 ± 227	1725 ± 396	1711 ± 113 *	1615 ± 221
**Uterus** (mg)	F	563 ± 77	711 ± 187	677 ± 268	623 ± 163
**Seminal Vesicle** (mg)	M	1565 ± 221	1721 ± 142	1711 ± 192	1652 ± 223

Values represent Mean ± S.D. (*n* = 10). * Significantly different from control (*p* < 0.05).

**Table 8:** Relative organ weights of male and female rats (Day 91/92).

Organ (Unit)	Sex	Control	107 U/kg	1070 U/kg	10,700 U/kg
**Brain** (g/100 g)	M	0.41 ± 0.02	0.42 ± 0.03	0.41 ± 0.04	0.40 ± 0.04
	F	0.70 ± 0.06	0.69 ± 0.04	0.68 ± 0.06	0.68 ± 0.05
**Heart** (g/100 g)	M	0.28 ± 0.02	0.29 ± 0.02	0.27 ± 0.02	0.27 ± 0.03
	F	0.32 ± 0.03	0.31 ± 0.02	0.31 ± 0.02	0.31 ± 0.03
**Liver** (g/100 g)	M	2.29 ± 0.19	2.38 ± 0.23	2.43 ± 0.20	2.55 ± 0.25
	F	2.36 ± 0.14	2.24 ± 0.11	2.23 ± 0.17 *	2.36 ± 0.11
**Kidneys** (g/100 g)	M	0.57 ± 0.03	0.59 ± 0.03	0.57 ± 0.03	0.59 ± 0.03
	F	0.63 ± 0.04	0.62 ± 0.05	0.61 ± 0.03	0.61 ± 0.03
**Spleen** (g/100 g)	M	0.15 ± 0.01	0.15 ± 0.01	0.14 ± 0.01	0.15 ± 0.01
	F	0.17 ± 0.02	0.16 ± 0.03	0.16 ± 0.02	0.18 ± 0.03
**Adrenals** (mg/100 g)	M	10.7 ± 1.6	10.1 ± 1.8	10.3 ± 1.4	11.2 ± 1.4
	F	21.8 ± 3.4	23.4 ± 3.4	19.9 ± 3.1	22.3 ± 4.4
**Testes** (g/100 g)	M	0.62 ± 0.05	0.63 ± 0.07	0.60 ± 0.06	0.63 ± 0.07
**Ovaries** (mg/100 g)	F	28.7 ± 5.2	30.1 ± 6.0	26.8 ± 3.8	31.4 ± 6.0
**Thyroid** (mg/100 g)	M	4.6 ± 0.7	5.3 ± 1.0	4.9 ± 0.9	5.2 ± 1.1
	F	7.9 ± 1.8	7.1 ± 1.4	7.6 ± 1.6	7.1 ± 1.6
**Pituitary** (mg/100 g)	M	2.6 ± 0.3	2.8 ± 0.5	2.6 ± 0.3	2.3 ± 0.3
	F	5.8 ± 1.1	5.1 ± 0.7	5.4 ± 1.0	5.2 ± 0.8
**Thymus** (mg/100 g)	M	66.2 ± 15.0	66.0 ± 19.9	60.5 ± 11.4	60.6 ± 17.5
	F	84.4 ± 24.3	85.5 ± 24.1	91.9 ± 21.3	93.3 ± 17.0
**Prostate** (mg/100 g)	M	263.3 ± 46.8	316.0 ± 65.5	298.5 ± 31.0	286.7 ± 45.3
**Uterus** (mg/100 g)	F	188.7 ± 34.0	226.2 ± 55.4	218.4 ± 76.5	201.2 ± 49.6
**Seminal Vesicle** (mg/100 g)	M	288.9 ± 34.9	318.5 ± 33.7	299.8 ± 45.1	291.6 ± 34.2

Values represent Mean ± S.D. (*n* = 10). Relative weights are organ weights divided by the body weight on the day of necropsy. * Significantly different from control (*p* < 0.05).

**Table 9:** Summary of revertant colony counts (Mean ± S.D.) in the bacterial reverse mutation test (modified IMF method for selected strains).

Strain	Metabolic Activation	Negative Control (Water)	Sumizyme PEG (107 U/plate)	Positive Control (a)
**TA100**	−S9	124 ± 6	142 ± 8	517 ± 24 (AF-2)
	+S9	153 ± 9	175 ± 2	578 ± 37 (2-AA)
**TA98**	−S9	18 ± 3	20 ± 5	244 ± 16 (AF-2)
	+S9	27 ± 3	22 ± 3	293 ± 5 (2-AA)
**TA1535**	−S9	13 ± 2 (b)	29 ± 5 (b)	637 (NaN3) (b)
	+S9	10 ± 2	13 ± 2	161 ± 16 (2-AA)
**TA1537**	−S9	17 (b)	22 (b)	723 (9-AA) (b)
	+S9	28 (b)	18 (b)	153 (2-AA) (b)
**WP2uvrA**	−S9	19 (b)	33 (b)	117 (AF-2) (b)
	+S9	29 (b)	32 (b)	796 (2-AA) (b)

(a) Positive controls: AF-2 = 2-(2-Furyl)-3-(5-nitro-2-furyl)acrylamide; NaN3 = Sodium azide; 9-AA = 9-Aminoacridine; 2-AA = 2-Aminoanthracene. (b) Data derived from standard pre-incubation method where modified IMF was not required or confirmatory data from standard method.

**Table 10:** Summary of the chromosome aberration test with Sumizyme PEG in CHL/IU cells (main and confirmative study).

Treatment Condition	S9	Study Phase	Dose (U/mL)	Relative Cell Growth (%)	Structural Aberrations (%)	Polyploid Cells (%)
**Short-term (6 h)**	−	Main	0	100.0	1.0	0.0
			107.0	101.0	6.5 *	0.0
		Confirmation	0	100.0	1.0	0.0
			74.9	100.0	2.5	0.0
			107.0	100.0	9.5 *	0.0
**Short-term (6 h)**	**+**	Main	0	100.0	1.0	0.0
			107.0	100.0	1.0	0.0
**Continuous (24 h)**	−	Main	0	100.0	0.5	0.5
			53.5	96.0	11.0 *	0.0
			107.0	94.0	21.5 *	0.0
		Confirmation	0	100.0	1.0	0.5
			74.9	93.0	18.0 *	0.0
			107.0	87.0	25.5 *	0.5
**Positive Controls**						
MMC (0.1 μg/mL)	−	6 h/24 h	—	—	19.5–24.5	0.0
CP (12 μg/mL)	+	6 h	—	—	42.0	0.0

* Significant increase compared to the negative control (*p* < 0.025).

**Table 11:** In vitro micronucleus test results in TK6 cells.

Treatment	Concentration (µg/mL)	RPD (%)	Osmolality (mOsm/kg)	pH	MN Frequency (%)
**3 h** −**S9**	0 (Control)	100.0	290	7.3	1.05
	23,900	101.4	338	7.2	1.05
**3 h +S9**	0 (Control)	100.0	288	7.3	1.15
	23,900	80.6	329	7.1	1.15
**24 h** −**S9**	0 (Control)	100.0	284	7.4	1.15
	15,933	94.7	308	7.3	1.35
	23,900	41.4	323	7.2	1.65

RPD: relative population doubling; MN: micronucleated cells.

**Table 12:** Full analytics of bone marrow micronucleus assay in male SD rats.

Group	Dose (U/kg)	IE/(IE + ME) Ratio (%)	MNIE/2000 IE (%)	Clinical Signs
**Negative Control**	0	52.7 ± 4.8	0.13 ± 0.06	Normal
**Sumizyme PEG**	2680	57.4 ± 2.0	0.20 ± 0.08	Normal
	5350	54.9 ± 4.0	0.11 ± 0.05	Normal
	10,700	57.5 ± 3.0	0.08 ± 0.03	Normal
**Positive Control (MMC)**	2 mg/kg	49.4 ± 7.4	3.69 ± 0.44 *	N/A

IE: immature erythrocyte; ME: mature erythrocyte; MNIE: micronucleated immature erythrocyte. * *p* < 0.025 (Kastenbaum and Bowman).

## Data Availability

The data presented in this study are available on request from the corresponding author.
